# A cross‐sectional study of the association between sleep disturbance profiles, unmet mental health or substance use needs, and presenteeism among United States activity‐duty service members using the 2018 health‐related behaviours survey (HRBS)

**DOI:** 10.1111/jsr.14477

**Published:** 2025-02-09

**Authors:** Teresa L. Russell, Darrell E. Singer, J. Kent Werner, James D. Mancuso, Anwar E. Ahmed

**Affiliations:** ^1^ Department of Preventive Medicine & Biostatistics Uniformed Services University of the Health Sciences Bethesda Maryland USA; ^2^ Department of Neurology Uniformed Services University of the Health Sciences Bethesda Maryland USA

**Keywords:** duty days, mental health conditions, military, presenteeism, productivity reduction, readiness, sleep disturbance, sleep medications, sleep quality, US military

## Abstract

Inadequate sleep, unmet mental health or substance use needs (unmet needs), and presenteeism are prevalent among military populations. This study aimed to cross‐sectionally determine the association between sleep disturbance profiles, unmet needs, and presenteeism in US active‐duty service members, both separately and combined. Data were collected from the 2018 Health‐Related Behaviours Survey. The response rate was 9.6%. Presenteeism was collected as the number of days (0–30) then collapsed for analysis. Latent class analysis (LCA) was used to classify service members into sleep disturbance profiles. Odds ratios and confidence intervals (CIs) were estimated by binary and ordinal logistic models. Approximately 21% of the 17,166 service members reported at least one presentee day (95% CI: 19.8%–21.8%). Persistent presenteeism was 13.6% (95% CI: 12.7–14.4%). Four sleep disturbance profiles were identified by LCA: (1) high sleep disturbance (reported in 22.5%), (2) short sleep duration (26%), (3) trouble sleeping (6.9%), and (4) none to slight sleep disturbance (reference, 44.6%). Female sex, being separated/divorced/widowed, short sleep duration, trouble sleeping, high sleep disturbance, unmet needs, and both unmet needs and inadequate sleep together were associated with higher odds of high presenteeism levels and persistent presenteeism. Bachelor's or higher educated, 25–34‐year‐old, Hispanic/Latinx, Officer, Air Force, and Coast Guard service members were associated with lower odds of high presenteeism levels and persistent presenteeism. Despite the decreasing trends between 2015 and 2018, the high prevalence of presenteeism presents a significant burden on work productivity and readiness that behavioural modification may alter.

## 1 INTRODUCTION

Presenteeism is defined as work or school days impaired due to mental or physical symptoms (Meadows et al., 2021). In contrast to absenteeism, where work or school days are missed, presenteeism describes days where individuals are present but their productivity is reduced due to their mental or physical health (Meadows et al., 2021). The prevalence of any presenteeism among active‐duty service members (ADSMs) in the United States (US) military was 24% in 2015, with 20% reporting persistent presenteeism in the past 30 days (Dunbar et al., 2022). Presenteeism can negatively impact worker health and result in both lost productivity and lower quality work, which is costly to both individual organisations and to the US economy overall (Silva‐Costa et al., 2020). The average annual cost of depression‐related presenteeism was estimated to be $5524 per person in the USA, for an aggregate cost of nearly $85 trillion each year in 2016 (equivalent to approximately $7236 and $110 trillion in 2024 dollars) (Evans‐Lacko & Knapp, 2016).

Reasons for presenteeism, absenteeism, and subsequent lost productivity include inadequate sleep and unmet mental health or substance use needs (unmet needs) (Alhola & Polo‐Kantola, 2007; Birnbaum et al., 2010; Buvik et al., 2018; Chattu et al., 2018; Itani et al., 2021; Joshi et al., 2023; Kessler et al., 2006; Parsley et al., 2022; Takano et al., 2024). Inadequate sleep can lead to impaired attention, visuomotor performance, alertness, reaction time, executive function, and decision‐making, which can all lead to presenteeism (Alhola & Polo‐Kantola, 2007; Chattu et al., 2018; Institute of Medicine Committee on Sleep Medicine and Research, 2006). Unmet mental health or substance use needs can lead to poor lifestyle behaviours (e.g. smoking, physical inactivity), the development of physical health conditions, and poor medication adherence, all of which can also lead to presenteeism (Goetzl et al., 2018). Active‐duty service members with mental health conditions are more likely to report presenteeism (Dunbar et al., 2022). A recent civilian survey similarly found that 45% of those reporting unmet mental health needs and 49% of those with unmet substance use needs reported issues at work (National Council for Mental Wellbeing, 2022). An estimated 45%–60% of adults in the USA suffer from comorbid mental health and substance use disorders (Danek et al., 2023; Reid & Palamar, 2022). Mental health or substance use disorders and inadequate sleep may also act together to further impact productivity (Joshi et al., 2023).

Inadequate sleep is nearly twice as prevalent in the military compared with the civilian population and the prevalence among ADSMs is increasing (56% in 2015 and 64% in 2018) (DOD, 2021; Meadows et al., 2018). Due to the demands of military training and work schedules, ADSMs' sleep is often disturbed by working inverted or elongated shifts, time zone changes, and exposure to stress and high noise levels (Good et al., 2020). Active‐duty service members report significantly higher rates of sleep deprivation during duty days (65%) than weekends (21%) (DOD, 2021). Unmet mental health or substance use needs are prevalent among ADSMs, with 57% of respondents reporting an unmet need in 2018 compared with 36% of respondents in 2015 (Dunbar et al., 2023; Meadows et al., 2018). Among ADSMs, a key reason for unmet needs is perceived professional risks as a consequence of seeking mental health or substance use care (Meadows et al., 2018; Meadows et al., 2021). Among ADSMs who responded to the HRBS, 34.2% of the 2018 sample and 34.5% of those with unmet mental health needs in 2015 believed that seeking mental health treatment would harm one's military career (Meadows et al., 2018; Meadows et al., 2021).

Active‐duty service members are particularly vulnerable to health risks and loss of productivity associated with inadequate sleep and unmet needs despite their higher baseline levels of health (DOD, 2021). In the military, presenteeism due to inadequate sleep and/or unmet needs can imperil the personal safety of ADSMs and impact military readiness, mission success, morale, and national security (Good et al., 2020). Presenteeism may be a better gauge of the impact of inadequate sleep and unmet needs than absenteeism or sick days in the ADSM population due to the requirements of military service. Sleep disturbance has been linked with both mental health and substance use and all are particularly pronounced among military personnel (DOD, 2021; Koffel et al., 2016; Scott et al., 2021). Few studies have estimated the prevalence of presenteeism and its link to sleep disturbance and unmet mental health or substance use needs in US ADSMs. We hypothesised that ADSMs with inadequate sleep and unmet mental health or substance use needs are associated with higher odds of presenteeism. In this study, we aimed to classify ADSMs into sleep disturbance profiles using latent class analysis (LCA) of sleep quality indicators and to identify any cross‐sectional associations between (1) sleep disturbance profiles and presenteeism, (2) unmet mental health or substance use needs and presenteeism, and (3) the combination of sleep and unmet health care needs and presenteeism among US ADSMs.

## 2 METHODS

### 2.1 Data source and sample

This study is a secondary analysis of the 2018 Health Related Behaviours Survey (HRBS) (Meadows et al., 2021). The HRBS is an anonymous, cross‐sectional online survey administered by the US Department of Defence (DOD) every 2 to 4 years (Meadows et al., 2021). Due to the COVID‐19 pandemic, the 2018 survey is the most recent HRBS at the time of this report. The purpose of the survey is to understand the health, health‐related behaviours, and well‐being of ADSMs and any potential impacts on productivity and readiness (Meadows et al., 2021).

The 2018 HRBS sampled all 1,357,219 ADSMs (as of September 2018) by service branch, pay grade, and sex (Meadows et al., 2021). A total of 199,996 ADSMs were invited to participate (Meadows et al., 2021). Of those invited, the final sample contained 17,166 surveys with usable data, for a weighted response rate of 9.6% (Meadows et al., 2021). Design and nonresponse weights were calculated and applied to account for unequal probabilities of selection (Meadows et al., 2021). The full 2018 HRBS methods are described in further detail elsewhere (Meadows et al., 2021).

### 2.2 Ethical considerations

This study received ethical approval from the Uniformed Services University Institutional Review Board (Protocol # DBS.2024.754). All elements of the 2018 HRBS were approved by the appropriate DOD and partner ethical review boards (Meadows et al., 2021).

### 2.3 Assessment of presenteeism

The primary outcome was presenteeism, defined as the average number of days present at work with performance compromised due to mental or physical symptoms in the past 30 days (Meadows et al., 2021). Presenteeism was assessed by the item “Thinking about any mental or physical symptoms you may have, on how many days in the PAST 30 DAYS, did you feel so impaired that, even though you went to work or school, your productivity was reduced?” (Meadows et al., 2021). The numerical answers from the respondents collapsed into four ordered categories: 0 days (none), 1 to 3 days, 4 to 14 days, and 15 to 30 days (high presenteeism levels). We also defined “persistent presenteeism” as 5 or more days of presenteeism in the past 30 days (Dunbar et al., 2022).

### 2.4 Assessment of unmet mental health or substance use needs

Perceived unmet mental health or substance use needs was assessed by the question “During the PAST 12 MONTHS, was there ever a time that you needed treatment for an emotional or mental health problem or for your use of alcohol or drugs but did not get it?” (yes/no) (Meadows et al., 2021).

### 2.5 Assessment of sleep behaviours

Sleep behaviours were measured on the 2018 HRBS using six indicators: lack of energy, sleep quantity, sleep quality, energy drink usage, and prescription sleep medications (Meadows et al., 2021). Latent class analysis was then performed to create sleep disturbance profiles from these six indicators.

Lack of energy was assessed by the item “In the past week, how much were you bothered by lack of energy because of poor sleep?” (Meadows et al., 2021). The four response options were collapsed into a dichotomous measure of slight to severe lack of energy, with respondents categorised into 1 = “yes” (2 = slightly bothered, 3 = moderately bothered or 4 = severely bothered) or 0 = “no” (1 = not bothered at all) (Meadows et al., 2021).

Sleep quantity was assessed by the question “On average, over the PAST 30 DAYS, how many hours of actual sleep do you get in a 24‐h period?” (Meadows et al., 2021). The numerical responses were categorised into a binary measure of sleeping below the recommended amount of 7 h, with options of 1 = “yes” (≤6 h) or 0 = “no” (≥7 h) (Meadows et al., 2021).

Sleep quality was assessed by the items (1) “During the PAST 30 DAYS, how would you rate your overall sleep quality?” and (2) “During the PAST 30 DAYS, how much have you been bothered by any of the following problems? Trouble sleeping?” (Meadows et al., 2021). The four response options for both were categorised into binary variables of (1) bad sleep quality and (2) trouble sleeping (Meadows et al., 2021). For bad sleep quality, respondents were categorised as 1 = “yes” (3 = fairly bad or 4 = very bad) or 0 = “no” (1 = very good or 2 = fairly good). For trouble sleeping, respondents were categorised as 1 = “yes” (2 = bothered a little bit, 3 = bothered a lot, 4 = very bad) or 0 = “no” (1 = not bothered at all) (Meadows et al., 2021).

Energy drink use was assessed by the question “During the PAST 30 DAYS, how often did you use the following TO HELP YOU STAY AWAKE? Energy drinks (e.g. Monster, Red Bull, Rockstar, 5‐Hour‐Energy)?” (Meadows et al., 2021). The five response options were categorised into a binary measure of energy drink use, with options of 1 = “yes” (2 = less than once a week, 3 = once or twice a week, 4 = three or more times a week, or 5 = daily) or 0 = “no” (never during the past 30 days) (Meadows et al., 2021).

Prescription sleep medications were assessed by the item “During the PAST 30 DAYS, how often did you take prescription or over‐the‐counter (OTC) medications TO HELP YOU SLEEP?” (Meadows et al., 2021). The four response options were categorised into a dichotomous variable of medication use, with options of 1 = “yes” (2 = less than once a week, 3 = once or twice a week, 4 = three or more times a week, 5 = daily) or 0 = “no” (1 = never during the past 30 days) (Meadows et al., 2021). For each of these binary variables, “no” was used as the reference category.

### 2.6 Sociodemographic and military characteristics

Covariables included in the study were sociodemographic and military characteristics. Sociodemographic factors included sex (*male [reference], female*), age (*17–24 years [reference], 25–34 years, ≥ 35 years*), education (*high school or less [reference], some college, Bachelor's Degree or more*), race (*White [reference], Black, Hispanic/Latinx, other*), marital status (*married [reference]; cohabitating; separate, divorced, or widowed*), and dependent children (*no [reference], yes*). Military factors included service branch (*Air Force, Army [reference], Marine Corps, Navy, Coast Guard*), and rank (*Enlisted [reference], Officer*).

### 2.7 Statistical analysis

Statistical analysis was performed using SAS® software version 9.4 (SAS Institute Inc., Cary, NC). Sampling weights were used in all analyses to correct for the nonresponse bias. Latent class analysis (LCA) was performed in Mplus (Version 8.8) to assign ADSMs to distinct sleep profiles (Muthén & Muthén, Los Angeles, CA). This model was based on the six sleep behaviours detailed above: lack of energy, sleep quantity, sleep quality, trouble sleeping, energy drink usage, and use of prescription sleep medications, with each indicator dichotomised into: yes (1) or no (0). The goodness of fit was compared across four solutions with different classes using information criterion statistics: the Akaike Information Criterion (AIC), the Bayesian Information Criterion (BIC), and the Adjusted Bayesian Information Criterion (Adjusted BIC). Weighted prevalence estimates of presenteeism (0 days (none), 1 to 3 days, 4 to 14 days, and 15 to 30 days) were compared across sleep disturbance profiles and demographic characteristics using the Rao‐Scott Chi‐square test (Table 1). Given the correlation between presenteeism and sleep and unmet health care needs, we created a new variable to assess the combination of sleep and unmet mental health and substance use needs (unmet needs) using four mutually exclusive categories: (1) no unmet needs and no inadequate sleep, (2) unmet needs only, (3) inadequate sleep only, and (4) both unmet needs and inadequate sleep. To prevent the inclusion of correlated exposures in a single model, we performed separate regression models to evaluate: (1) unmet needs controlling for demographic characteristics, (2) sleep profiles controlling for demographic characteristics, and (3) the combined effect of unmet needs and inadequate sleep (four mutually exclusive categories) controlling for demographic characteristics. Using this structure, the cumulative odds ratio of high presenteeism levels for each exposure was estimated separately using weighted ordinal regression models (Table 2) and weighted binary logistic regression for persistent presenteeism was performed for each exposure separately (Table 3).

**TABLE 1:** Prevalence estimates of presenteeism by demographic and sleep profiles.

	Duty days impaired due to mental or physical symptoms, past 30 days (presenteeism)										
	Overall		0 days (none)		1 to 3 days		4 to 14 days		15 to 30 days		*p*
	*n*	%	*n*	%	*n*	%	*n*	%	*n*	%	
Total	17,166	100.0	13,591	79.2	1116	5.9	1473	8.1	986	6.7	
Sex											<0.0001
Female	5353	16.7	3869	71.1	483	8.9	612	11.2	389	8.8	
Male	11,813	83.3	9722	80.8	633	5.3	861	7.5	597	6.3	
Age											0.007
17–24 years	3642	37.8	2866	77.8	212	6.0	329	8.9	235	7.4	
25–34 years	6467	39.9	5208	80.9	414	5.5	491	7.1	354	6.6	
≥35 years	7057	22.3	5517	78.6	490	6.7	653	8.8	397	5.9	
Education											<0.0001
High school or less	7990	65.2	6255	78.0	481	5.9	704	8.2	550	7.9	
Some college	2625	13.0	2040	79.3	174	5.5	247	9.1	164	6.1	
Bachelor's degree or more	6301	21.9	5080	82.6	445	6.3	507	7.1	269	4.0	
Race											0.033
Black	2226	16.3	1739	79.1	153	6.1	183	7.5	151	7.3	
Hispanic/Latinx	2459	16.1	1989	80.9	169	6.8	170	7.6	131	4.7	
Other	1747	9.6	1404	81.2	109	5.2	132	7.0	102	6.6	
White	10,666	58.0	8404	78.6	680	5.7	985	8.6	597	7.1	
Marital status											<0.0001
Married	10,776	53.8	8650	79.9	680	5.6	873	7.8	573	6.7	
Cohabiting	1042	7.8	783	74.5	66	7.4	124	12.5	69	5.6	
Separated, divorced, or widowed	1284	6.3	911	71.6	93	5.1	169	13.1	111	10.2	
Never married	4064	32.1	3247	80.7	277	6.3	307	6.7	233	6.3	
Dependent children											0.79
No	8629	60.3	6841	78.8	567	6.1	734	8.3	487	6.8	
Yes	8537	39.7	6750	79.8	549	5.8	739	7.9	499	6.6	
Service branch											<0.0001
Air force	5579	24.1	4647	85.0	323	5.0	371	5.9	238	4.1	
Army	3646	34.5	2815	78.7	230	4.9	347	8.8	254	7.6	
Marine corps	2569	13.9	1957	76.8	149	6.0	272	10.0	191	7.2	
Navy	3675	24.4	2797	75.1	303	8.3	343	8.5	232	8.1	
US Coast Guard	1697	3.2	1375	82.5	111	5.8	140	7.1	71	4.7	
Rank											<0.0001
Enlisted	12,154	83.5	9544	78.4	735	5.8	1071	8.4	804	7.4	
Officer	5012	16.5	4047	83.0	381	6.8	402	6.8	182	3.4	
Unmet needs^a^											<0.0001
Yes	16,052	93.2	13,183	82.6	976	5.5	1184	6.9	709	5.0	
No	1114	6.8	408	32.8	140	12.1	289	25.3	277	29.9	
Sleep profiles											<0.0001
High sleep disturbance (Severe)	3702	22.5	2050	53.9	345	8.3	678	17.9	629	19.9	
Sleep duration	4270	26.0	3435	80.9	297	6.5	354	7.7	184	5.0	
Trouble sleep	1304	6.9	934	72.5	132	10.3	173	12.2	65	5.0	
None to slight sleep disturbance	7890	44.6	7172	92.0	342	3.7	268	2.9	108	1.3	
Combination of unmet needs^a^ and inadequate sleep^b^											<0.0001
No unmet needs^a^ and inadequate sleep^b^	7713	43.6	7076	92.9	313	3.5	235	2.4	89	1.1	
Unmet needs^a^ only	177	1.0	96	52.8	29	13.6	33	22.1	19	11.5	
Inadequate sleep^b^ only	8339	49.6	6107	73.5	663	7.2	949	10.8	620	8.5	
Both unmet needs^a^ & inadequate sleep^b^	937	5.8	312	29.4	111	11.8	256	25.8	258	32.9	

^a^ Unmet needs include mental health or substance use (alcohol or drugs) needs.

^b^ Inadequate sleep includes sleep classes 1–3 vs. reference of class 4 (none to slight sleep disturbance).

**TABLE 2:** Unadjusted and adjusted ordinal logistic regression of factors associated with high presenteeism levels.

	Duty days impaired due to mental or physical symptoms, past 30 days (presenteeism)					
	0 days (none), 1 to 3 days, 4 to 14 days, and 15 to 30 days					
	Unadjusted			Adjusted		
	OR	LCL	UCL	aOR	LCL	UCL
Sex						
Female	**1.666**	**1.485**	**1.868**	**1.757**	**1.553**	**1.987**
Male	1.000			1.000		
Age						
17–24 years	1.066	0.927	1.227	0.882	0.713	1.091
25–34 years	**0.887**	**0.790**	**0.996**	**0.802**	**0.704**	**0.913**
≥35 years	1.000			1.000		
Education						
High school or less	1.000			1.000		
Some college	0.915	0.788	1.063	0.961	0.814	1.134
Bachelor's degree or more	**0.723**	**0.644**	**0.811**	**0.742**	**0.626**	**0.880**
Race						
Black	0.970	0.809	1.163	0.842	0.695	1.020
Hispanic/Latinx	0.843	0.709	1.001	**0.745**	**0.624**	**0.890**
Other	0.854	0.710	1.028	**0.759**	**0.626**	**0.920**
White	1.000			1.000		
Marital status						
Married	1.000			1.000		
Cohabiting	**1.315**	**1.048**	**1.649**	1.143	0.889	1.469
Separated, divorced, or widowed	**1.612**	**1.296**	**2.006**	**1.383**	**1.122**	**1.703**
Never married	0.942	0.811	1.093	**0.806**	**0.662**	**0.982**
Dependent children						
No	1.000			1.000		
Yes	0.946	0.841	1.065	0.893	0.750	1.063
Service branch						
Air force	**0.638**	**0.549**	**0.741**	**0.627**	**0.540**	**0.729**
Army	1.000			1.000		
Marine Corps	1.101	0.916	1.324	1.110	0.906	1.360
Navy	**1.193**	**1.001**	**1.422**	1.169	0.975	1.402
US Coast Guard	**0.765**	**0.626**	**0.936**	**0.705**	**0.572**	**0.869**
Rank						
Enlisted	1.000			1.000		
Officer	**0.717**	**0.641**	**0.803**	0.903	0.768	1.062
Unmet needs^a^, ^c^						
Yes	**8.995**	**7.418**	**10.907**	**7.910**	**6.516**	**9.603**
No	1.000			1.000		
Sleep profiles^c^						
High sleep disturbance	**10.723**	**9.143**	**12.576**	**10.073**	**8.552**	**11.865**
Sleep duration	**2.748**	**2.316**	**3.262**	**2.733**	**2.294**	**3.257**
Trouble sleep	**4.226**	**3.388**	**5.270**	**3.977**	**3.182**	**4.971**
None to slight sleep disturbance	1.000			1.000		
Combination of unmet needs^a^ and inadequate sleep^b^, ^c^						
No unmet needs^a^ and inadequate sleep^b^	1.000			1.000		
Unmet needs^a^ only	**10.841**	**6.607**	**17.786**	**10.085**	**6.147**	**16.545**
Inadequate sleep^b^ only	**4.858**	**4.194**	**5.626**	**4.709**	**4.056**	**5.466**
Both unmet needs^a^ & inadequate sleep^b^	**29.924**	**23.835**	**37.569**	**26.306**	**20.900**	**33.110**

*Note*: Boldface indicates statistical significance (*p* = 0.05).

^a^ Unmet needs include mental health or substance use (alcohol or drugs) needs.

^b^ Inadequate sleep includes sleep classes 1–3 vs. reference of class 4 (none to slight sleep disturbance).

^c^ Each exposure variable is adjusted for demographic variables in the model.

**TABLE 3:** Unadjusted and adjusted binary logistic regression of factors associated with persistent presenteeism.

	Unadjusted			Adjusted		
	OR	LCL	UCL	aOR	LCL	UCL
Sex						
Female	**1.572**	**1.373**	**1.799**	**1.679**	**1.449**	**1.944**
Male	1.000			1.000		
Age						
17–24 years	1.092	0.925	1.290	0.914	0.711	1.174
25–34 years	0.926	0.807	1.063	**0.842**	**0.721**	**0.982**
≥35 years	1.000			1.000		
Education						
High school or less	1.000			1.000		
Some college	0.974	0.815	1.164	1.004	0.821	1.228
Bachelor's degree or more	**0.637**	**0.553**	**0.733**	**0.697**	**0.569**	**0.853**
Race						
Black	0.946	0.763	1.172	**0.795**	**0.634**	**0.998**
Hispanic/Latinx	**0.733**	**0.588**	**0.913**	**0.631**	**0.503**	**0.790**
Other	0.870	0.698	1.085	**0.786**	**0.625**	**0.987**
White	1.000			1.000		
Marital status						
Married	1.000			1.000		
Cohabiting	1.212	0.921	1.593	1.048	0.770	1.426
Separated, divorced, or widowed	**1.610**	**1.282**	**2.023**	1.492	1.181	1.884
Never married	**0.883**	**0.736**	**1.060**	**0.751**	**0.591**	**0.954**
Dependent children						
No	1.000			1.000		
Yes	0.985	0.855	1.135	0.892	0.724	1.100
Service branch						
Air force	**0.569**	**0.477**	**0.679**	**0.549**	**0.459**	**0.656**
Army	1.000			1.000		
Marine corps	1.063	0.860	1.313	1.061	0.837	1.344
Navy	1.041	0.847	1.279	1.004	0.810	1.245
US Coast Guard	**0.652**	**0.510**	**0.835**	**0.579**	**0.446**	**0.751**
Rank						
Enlisted	1.000			1.000		
Officer	**0.592**	**0.515**	**0.681**	**0.774**	**0.637**	**0.941**
Unmet needs^a^, ^c^						
Yes	**9.641**	**7.791**	**11.93**	**8.445**	**6.781**	**10.52**
No	1.000			1.000		
Sleep profiles^c^						
High sleep disturbance	**14.182**	**11.451**	**17.564**	**13.033**	**10.463**	**16.233**
Sleep duration	**3.531**	**2.792**	**4.465**	**3.425**	**2.696**	**4.352**
Trouble sleep	**5.096**	**3.789**	**6.852**	**4.685**	**3.468**	**6.329**
None to slight sleep disturbance	1.000			1.000		
Combination of unmet needs^a^ and inadequate sleep^b^, ^c^						
No unmet needs^a^ and inadequate sleep^b^	1.000			1.000		
Unmet needs^a^ only	**43.32**	**32.72**	**57.37**	**14.57**	**7.699**	**27.57**
Inadequate sleep^b^ only	**6.899**	**5.604**	**8.493**	**6.557**	**5.305**	**8.104**
Both unmet needs^a^ & inadequate sleep^b^	**43.32**	**32.72**	**57.37**	**37.25**	**27.94**	**49.66**

*Note*: Boldface indicates statistical significance (*p* = 0.05).

^a^ Unmet needs include mental health or substance use (alcohol or drugs) needs.

^b^ Inadequate sleep includes sleep classes 1–3 vs reference of class 4 (none to slight sleep disturbance).

^c^ Each exposure variable is adjusted for demographic variables in the model.

## 3 RESULTS

Approximately 21% of the 17,166 ADSMs reported at least one presentee day (95% confidence interval (CI): 19.8%–21.8%), with servicewomen more significantly impacted (28.9% vs. 19.2%, *p* < 0.0001) compared with servicemen. Overall, 6% reported 1–3 days, 8% reported 4–14 days, and 7% reported 15–30 days of presenteeism (Table 1). The prevalence of persistent presenteeism was 13.6% (95% CI: 12.7–14.4%), with an average of 2.2 (standard error = 0.1) presentee days in the past 30 days. Presentee days significantly differed among all covariables (all *p* < 0.05) except dependent children: 0 days (78.8% no vs 79.8% yes), 1 to 3 days (6.1% no vs 5.8% yes), 4 to 14 days (8.3% no vs 7.9% yes), 15 to 30 days (6.8% no vs 6.6% yes), (*p* = 0.790).

**FIGURE 1:**
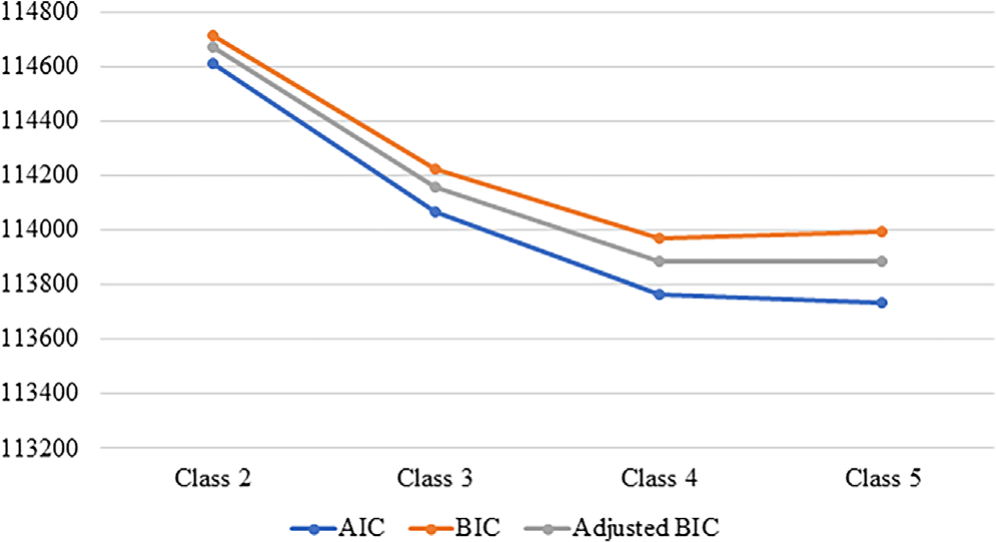
Goodness of fit across different number of classes.

**FIGURE 2:**
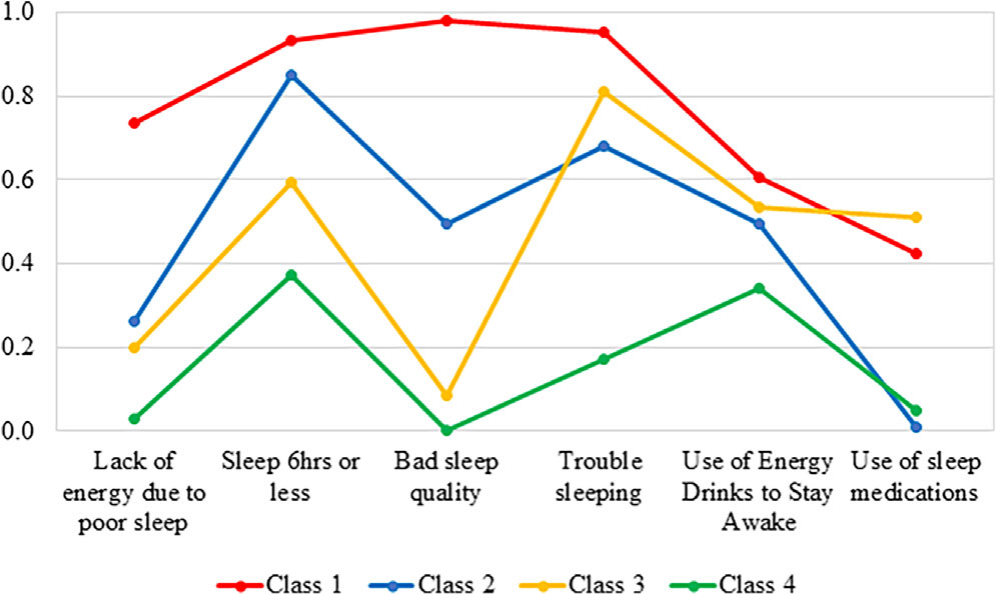
Conditional probabilities of “yes” response. Class 1: High sleep disturbance (Severe) (22.5%), Class 2: Short sleep duration (26.0%), Class 3: Trouble sleeping (6.9%), and Class 4: None to slight sleep disturbance (44.6%).

Latent class analysis was used to classify ADSMs into four different sleep disturbance profiles. Figure 1 displays the goodness of fit across four models: two‐, three‐, four‐, and five‐class solutions. The four‐class solution had a good fit as the BIC across value was lower than the other solutions. According to the probability of endorsing each sleep behaviour given class membership, the sleep disturbance profiles were labelled as follows (Figure 2). Class 1 denoted high or severe sleep disturbance and was reported in 22.5% of ADSMs. Class 2 denoted short sleep duration and was reported by 26%. Class 3 denoted trouble sleeping and was reported by 6.9%. Class 4 denoted none to slight sleep disturbance and was reported in 44.6%. Class 4 was used as the reference category.

The results of bivariate analyses using ordered and binary logistic regression models to estimate the unadjusted cumulative odds ratio of high presenteeism levels (Table 2) and the unadjusted odds ratio of persistent presenteeism (Table 3) highlight several significant associations. ADSMs with high sleep disturbance, short sleep duration, and trouble sleeping had significantly higher odds of high presenteeism levels and persistent presenteeism compared with ADSMs with none to slight sleep disturbance. Those with unmet mental health or substance use needs also had significantly higher odds of high presenteeism levels and persistent presenteeism, compared with those with no unmet needs. In the combined exposure analysis, unmet needs only, inadequate sleep only, and both unmet needs and inadequate sleep had significantly higher odds of high presenteeism levels and persistent presenteeism, compared with those with no unmet needs and no inadequate sleep.

**FIGURE 3:**
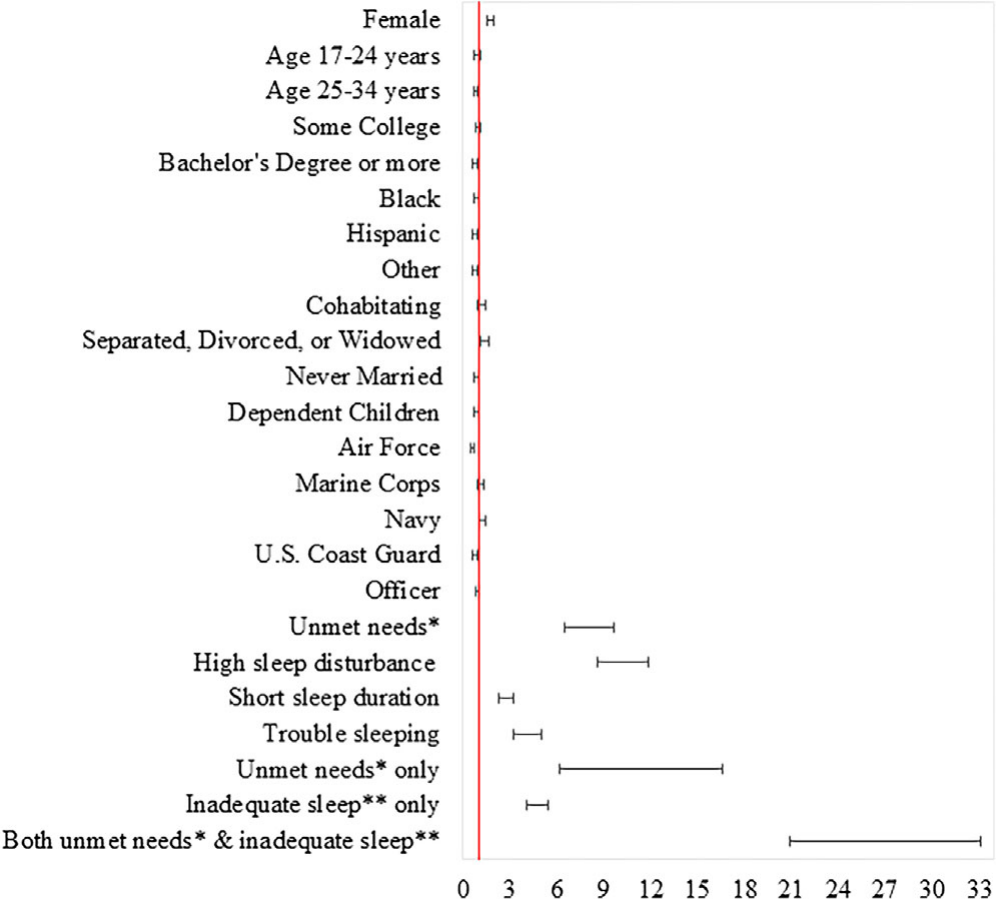
Forrest plot of adjusted analysis of factors associated with high presenteeism levels. *Unmet needs include mental health or substance use (alcohol or drugs) needs. **Inadequate sleep includes sleep classes 1–3 versus reference of class 4 (none to slight sleep disturbance). Each exposure variable is adjusted for demographic variables in the model.

**FIGURE 4:**
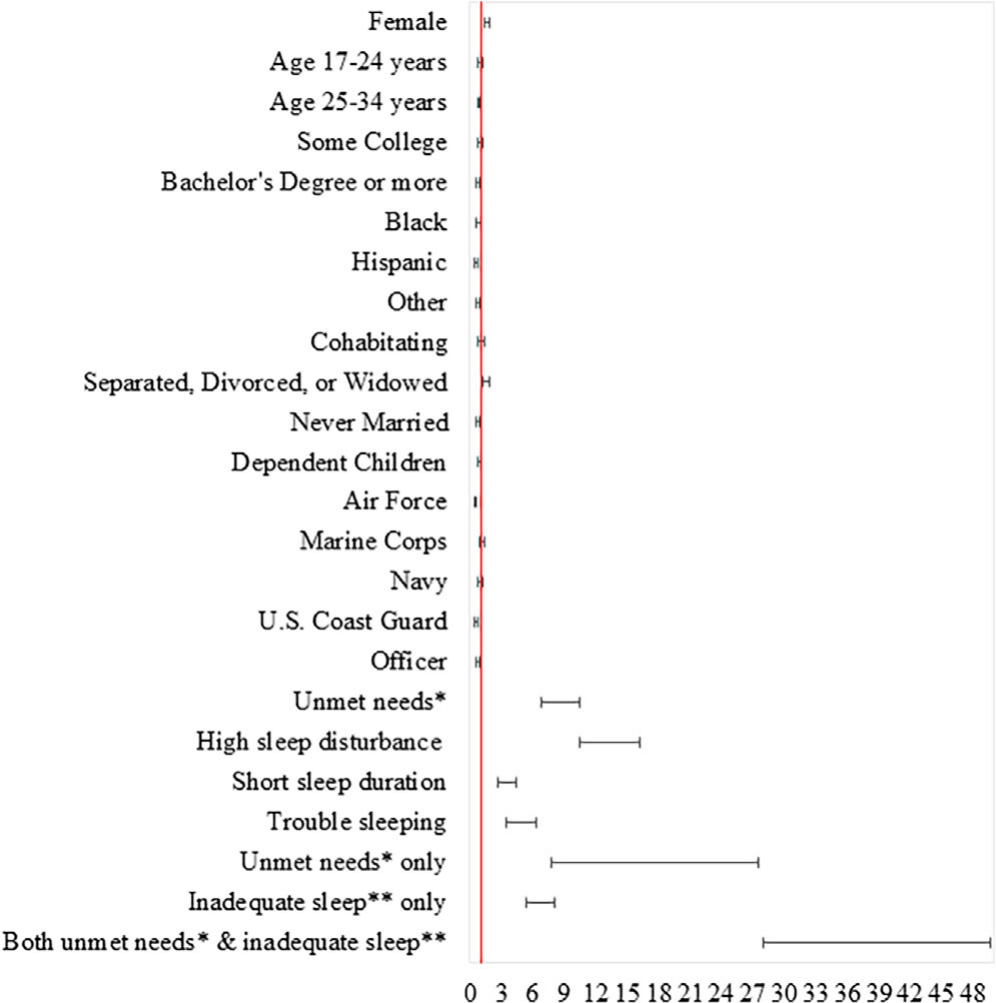
Forrest plot of adjusted analysis of factors associated with persistent presenteeism (reference = no). *Unmet needs include mental health or substance use (alcohol or drugs) needs. **Inadequate sleep includes sleep classes 1–3 vs. reference of class 4 (none to slight sleep disturbance). Each exposure variable is adjusted for demographic variables in the model.

On adjusted analysis, ADSMs with high sleep disturbance (aOR = 10.073, 95% CI: 8.552–11.865; aOR = 13.033, 95% CI: 10.463–16.233), short sleep duration (aOR = 2.733, 95% CI: 2.294–3.257; aOR = 3.425, 95% CI: 2.696–4.352), and trouble sleeping (aOR = 3.977, 95% CI: 3.182–4.971; aOR = 4.685, 95% CI: 3.468–6.329) had significantly higher odds of high presenteeism levels and persistent presenteeism compared with those with none to slight sleep disturbance, respectively (Tables 2 and 3 and Figures 3 and 4). ADSMs with unmet mental health or substance use needs had significantly higher odds of high presenteeism levels (aOR = 7.910, 95% CI: 6.516–9.603) and persistent presenteeism (aOR = 8.445, 95% CI: 6.781–10.518), compared with those with no unmet needs.

We also examined the combined effect (four mutually exclusive categories) of unmet mental health or substance use needs and sleep profiles on the odds of high presenteeism levels and persistent presenteeism. Compared with ADSMs with no unmet needs and no inadequate sleep, ADSMs with unmet needs only (aOR = 10.085, 95% CI: 6.147–16.545; aOR = 14.568, 95% CI: 7.699–27.566), inadequate sleep only (aOR = 4.709, 95% CI: 4.056–5.466; aOR = 6.557, 95% CI: 5.305–8.104), and both unmet needs and inadequate sleep (aOR = 26.306, 95% CI: 20.900–33.110; aOR = 37.253, 95% CI: 27.943–49.664) had significantly higher odds of high presenteeism levels and persistent presenteeism, respectively.

Female sex and separated, divorced, or widowed were associated with higher odds of high presenteeism levels and persistent presenteeism. Bachelor's or higher educated, 25–34‐year‐old, Hispanic/Latinx, Officer, and Air Force, and Coast Guard ADSMs had lower odds of high presenteeism levels and persistent presenteeism.

## 4 DISCUSSION

In the largest DOD survey of ADSMs, over one‐fifth (21.0%) reported any presentee day in the past month and 13.6% reported persistent presenteeism. The overall prevalence estimates have significantly decreased over a 3‐year period (2015–2018) (Dunbar et al., 2022). Our study found that the proportion of ADSMs with any presentee day decreased from 24% in 2015 to 21% in 2018 (95% CI: 19.8%–21.8%) and persistent presenteeism decreased from 20% in 2015 to 13.6% in 2018 (95% CI: 12.7–14.4%) (Dunbar et al., 2022).

Four sleep disturbance classes were identified: (1) high sleep disturbance, (2) short sleep duration, (3) trouble sleeping, and (4) none to slight sleep disturbance, which was used as the reference category. We found a high overall prevalence of inadequate sleep (defined as any class of sleep disturbance, class 1 to 3) of 55.4%, consistent with the 57.8% (95% CI: 49.9%–65.5%) prevalence of poor sleep quality in ADSMs found previously (Bai et al., 2023). We identified a strong association between trouble sleeping and both high presenteeism levels and persistent presenteeism. It is crucial to differentiate between trouble sleeping and short sleep duration. Short sleep duration can result from lifestyle choices or work schedules, whereas trouble sleeping or difficulty falling asleep is a clinical symptom of insomnia (Bastien et al., 2001). Insomnia can be a chronic condition that may require different types of intervention such as addressing the underlying causes, including medical or mental health conditions (Carroll et al., 2015; Hertenstein et al., 2019). This study found significantly increased odds of presenteeism among ADSMs with short sleep duration, trouble sleeping, and especially with high sleep disturbance and the combination of sleep and unmet mental health or substance use needs, in accordance with previous work (Dunbar et al., 2022; Itani et al., 2021; Joshi et al., 2023; Takano et al., 2024). The prevalence and characteristics of sleep disturbance among the military population and its impacts on military readiness have been described in detail (DOD, 2021). However, the relationship between inadequate sleep, unmet needs, and presenteeism has not been well‐studied in the military population previously. This study's findings are therefore key in adding specific data relating complex sleep disturbance profiles, unmet mental health and substance use needs (both independently and in combination), and presenteeism among the US active‐duty component.

In this study, unmet needs for mental health or substance use services among ADSMs was also significantly and independently associated with higher odds of presenteeism in ADSMs. The link between mental health or substance use needs and increased risk for presenteeism found in this study is consistent with previous work in the civilian and military populations (Dunbar et al., 2022; Evans‐Lacko & Knapp, 2016; Goetzel et al., 2018; Kessler et al., 2006; National Council for Mental Wellbeing, 2022). Mental health conditions have been linked with greater absenteeism and presenteeism in the ADSM population using data from the 2015 HRBS (Dunbar et al., 2022). This study found that these associations persisted in 2018. While existing literature largely focusses on inadequate sleep or unmet needs impacting presenteeism, this study highlights the significant combined (between additive and multiplicative) effect on presenteeism and persistent presenteeism.

Female sex has been reported as a risk factor for presenteeism in several previous studies (Goto et al., 2020; Merrill et al., 2012). This study found that female sex was significantly associated with increased odds of presenteeism, compared with male sex. A previous study found that women may feel more pressure to engage in extra‐role behaviours (going above and beyond at work) due to normative influences (pressure from society, organisation, and colleagues) compared with men (Luksyte et al., 2023). This behaviour can detrimentally impact health and may relate to the higher odds of presenteeism among females found in this study. Additionally, females are more likely to be the primary caregiver responsible for child and family care, even when both spouses are employed full‐time and demonstrate equal competency (Jones et al., 2022; Pew Research Center, 2015; Schoppe‐Sullivan et al., 2021). Additional gender norms and social pressures may impact the mental load of female ADSMs leading to the higher levels of presenteeism reported in this study. Finally, separated, divorced, or widowed ADSMs' significantly higher odds of presenteeism and never married ADSMs' significantly lower odds of presenteeism may be interrelated with mental health or substance use and sleep according to previous studies (Chen et al., 2015; Uecker, 2012).

Previous work found that people of minority race/ethnicity had lower rates of presenteeism than people of non‐minority race/ethnicity, with no significant differences between race/ethnicity groups (Warren et al., 2011). This is consistent with this study's finding that Hispanic/Latinx ADSMs had significantly lower odds of presenteeism compared with White ADSMs. However, another study found that non‐citizen, naturalised, and US‐born Latinx workers were significantly more likely to have unmet needs for paid leave compared with US‐born White respondents, which may lead to increased presenteeism behaviours due to being unable to take needed leave (Haro‐Ramos & Bacong, 2023). Presenteeism among Hispanic/Latinx ADSMs may also be increased due to perceived everyday discrimination in this subpopulation compared with White ADSMs (Deng et al., 2020). The differences found in presenteeism between racial/ethnic groups may be due to the availability of time off or differences in experiences of everyday discrimination among military populations.

Evans‐Lacko and Knapp (2016) found that a higher education level was associated with significantly higher odds of presenteeism. Other studies found no significant increased prevalence of presenteeism among those with a Bachelor's degree or higher (Haro‐Ramos & Bacong, 2023; Merrill et al., 2012). This study found that higher education was inversely related to presenteeism. ADSMs with a Bachelor's degree or higher had significantly lower odds of presenteeism, compared with those with a high school education or less. Differences between this study's findings and that of previous work may be due to the nature of work done by ADSMs with this level of education compared with civilian workers.

The significantly lower presenteeism among Air Force and Coast Guard compared with Army ADSMs found in this study may be due to differences in the nature of service or quality of life of ADSMs in the different branches. Work–life balance, cultural norms, and stigma related to taking days off may also differ between branches and contribute to the variations in presenteeism found. Similarly, officers may have different job expectations and nature of service than enlisted ADSMs, resulting in the significantly lower odds of presenteeism found among officers in this study. However, due to the general dearth of data on reasons for presenteeism in the military, it is unclear from the existing literature why the significant differences in presenteeism between service branches and between officer and enlisted personnel found in this study may exist.

Due to the pronounced link found between sleep and presenteeism and unmet mental health service needs and presenteeism, future work should look to longitudinally track changes in sleep disturbance and mental health utilisation over time among ADSMs. If future iterations of the HRBS use similar methods that permit comparison of cohorts across HRBS survey years, reproduction of the methods used in this study could be used across multiple years of data to showcase cohort‐level changes over time. However, as the HRBS is an anonymous survey, it would not allow for longitudinal tracking of individual‐level changes over time. Individual‐level data could allow for consideration of more specific factors including medical and social history which may impact sleep, mental health utilisation, and presenteeism. Future research could also collect additional details on reasons for presenteeism, as done by Merrill et al. (2012), which may help to target interventions to mitigate presenteeism within the active‐duty component. As found in this study, sleep and mental health care seeking behaviours may be key targets of behavioural interventions to improve military presenteeism. Policies that protect healthy sleep patterns as much as possible among ADSMs should be prioritised across all branches. Existing prevention programmes and policies enabling mental health care utilisation, including DODIs 6490.04 and 6490.08, should be reviewed and optimised to address the large burden of unmet mental health or substance use needs. This is in line with the 2024 National Defence Authorization Act, which continued to focus on advancing policy and research on military mental health care programmes and services to support ADSMs, retirees, and beneficiaries (Mendez, 2024). This study highlighted the importance of sleep behaviours and mental health care utilisation on work productivity and presenteeism and presents research and policy opportunities for potential improvement in US military productivity and readiness.

### 4.1 Limitations

This study was limited by the cross‐sectional and self‐reported survey‐based nature of the data on which this study did secondary analysis. Cross‐sectional design limits causal inference. Self‐reported measures are subject to recall bias. Social desirability bias may also impact responses, particularly among the ADSM population. The lack of individual‐level data in the anonymous HRBS data set may have resulted in residual confounding by unmeasured variables. Due to differences in survey questions and sampling methods across years, it is not possible to examine trends over time using the currently collected HRBS data. The response rate for ADSMs was 8.6% unweighted or 9.6% weighted, which is relatively low for a survey study and may mean the results are impacted by selection bias (Meadows et al., 2021). However, the survey weights are designed to minimise selection bias and increase the representativeness of the sample to the entire ADSM population (Meadows et al., 2021). An additional limitation of the HRBS survey design is a lack of granularity on certain demographics used as covariables in this analysis. Collapsing multiple races in the “Other race” subgroup leads to an inability to fully consider impacts among these racial groups. Additionally, having information only on biological sex without detail on gender identity and presentation may have led to incomplete consideration of gendered expectations. The HRBS dataset additionally did not have an adequate variable or set of variables that could serve as a proxy of measure for morale or leadership quality, a key factor potentially related to presenteeism. Future HRBS surveys may consider adding specific indicators of leadership quality or morale to better evaluate their effects on presenteeism.

Despite these limitations, this study's large sample size and well‐controlled, complex survey design are designed for the generalisability of these results to the overall US ADSM population (Meadows et al., 2021). The large sample size also allowed for testing of a large number of covariables to get a clearer picture of high‐risk groups for presenteeism. Utilisation of LCA to generate complex, multidimensional sleep disturbance profiles allowed for a more rigorous evaluation of sleep behaviours than individual survey measures alone.

## 5 CONCLUSION

Despite the decreasing trends in estimates between 2015 and 2018, the high prevalence of ADSMs reporting at least one presentee day and persistent presenteeism may present a significant burden on work productivity and readiness. Possible higher‐risk groups to target include female, enlisted, and separated/divorced/widowed ADSMs for high presenteeism levels and persistent presenteeism. For both high presenteeism levels and persistent presenteeism, ADSMs with short sleep duration, trouble sleeping, high sleep disturbance, unmet mental health or substance use needs, and especially those with both inadequate sleep and unmet needs are key high‐risk groups to target with interventions. Sleep and mental health or substance use care‐seeking behaviours may be modifiable factors to reduce presenteeism among ADSMs and to improve readiness. Longitudinal trajectories of inadequate sleep, unmet mental health or substance use need, and presenteeism may provide important implications for identifying targeted interventions.

## DISCLAIMERS

The contents of this publication are the sole responsibility of the authors and do not necessarily reflect the views, opinions, or policies of Uniformed Services University of the Health Sciences (USU), the Department of Defence, or the Departments of the Army, Navy, or Air Force. Mention of trade names, commercial products, or organisations does not imply endorsement by the US Government. This work was prepared by an employee of the US Government as part of the individual's official duties and therefore is in the public domain and does not possess copyright protection (however, as a courtesy it is requested that USU and the author be given an appropriate acknowledgement).

## AUTHOR CONTRIBUTIONS

**Teresa L. Russell:** Validation; writing – original draft. **Darrell E. Singer:** Supervision; validation; writing – review and editing. **J. Kent Werner Jr.:** Writing – review and editing; visualization. **James D. Mancuso:** Supervision; validation; writing – review and editing. **Anwar E. Ahmed:** Conceptualization; writing – original draft; writing – review and editing; supervision; formal analysis.

## FUNDING INFORMATION

The authors received no financial support for this research.

## CONFLICT OF INTEREST STATEMENT

The authors declare no competing interests.

## Data Availability

The data that support the findings of this study are available from RAND and DOD but restrictions apply to the availability of these data, which were used under licence for the current study, and so are not publicly available.
